# Supplementary material for: Analysis and prediction of the major fatty acids in vegetable oils using dielectric spectroscopy at 5–30 MHz
Source: PLoS One. 2022 May 26;17(5):e0268827. doi: 10.1371/journal.pone.0268827 (PMC9135300; doi:10.1371/journal.pone.0268827)
Supplement: S1 File — (DOCX) [file pone.0268827.s001.docx]

**Supporting Information**

**Table 5. Prediction evaluation of the PCR and PLS analysis.**

| Fatty acids | PLS Model | | | | PCR Analysis | | | |
| --- | --- | --- | --- | --- | --- | --- | --- | --- |
|  | No. Latent Variables | RMSECV (%) | MAPE (%) | R^2^ | No. Principal Component | RMSECV (%) | MAPE (%) | R^2^ |
| C18:1 | 5 | 9.19 | 18.08 | 0.84 | 5 | 9.21 | 18.22 | 0.84 |
| C18:2 | 5 | 11.23 | 23.58 | 0.77 | 5 | 11.19 | 23.50 | 0.77 |
| C18:3 | 5 | 5.28 | 154.88 | 0.40 | 5 | 5.29 | 157.32 | 0.39 |
| SFA | 5 | 0.18 | 55.14 | 0.55 | 5 | 0.18 | 56.36 | 0.55 |
| MUFA | 5 | 9.17 | 17.92 | 0.84 | 5 | 9.19 | 18.05 | 0.84 |
| PUFA | 5 | 9.24 | 18.85 | 0.84 | 5 | 9.26 | 18.90 | 0.84 |


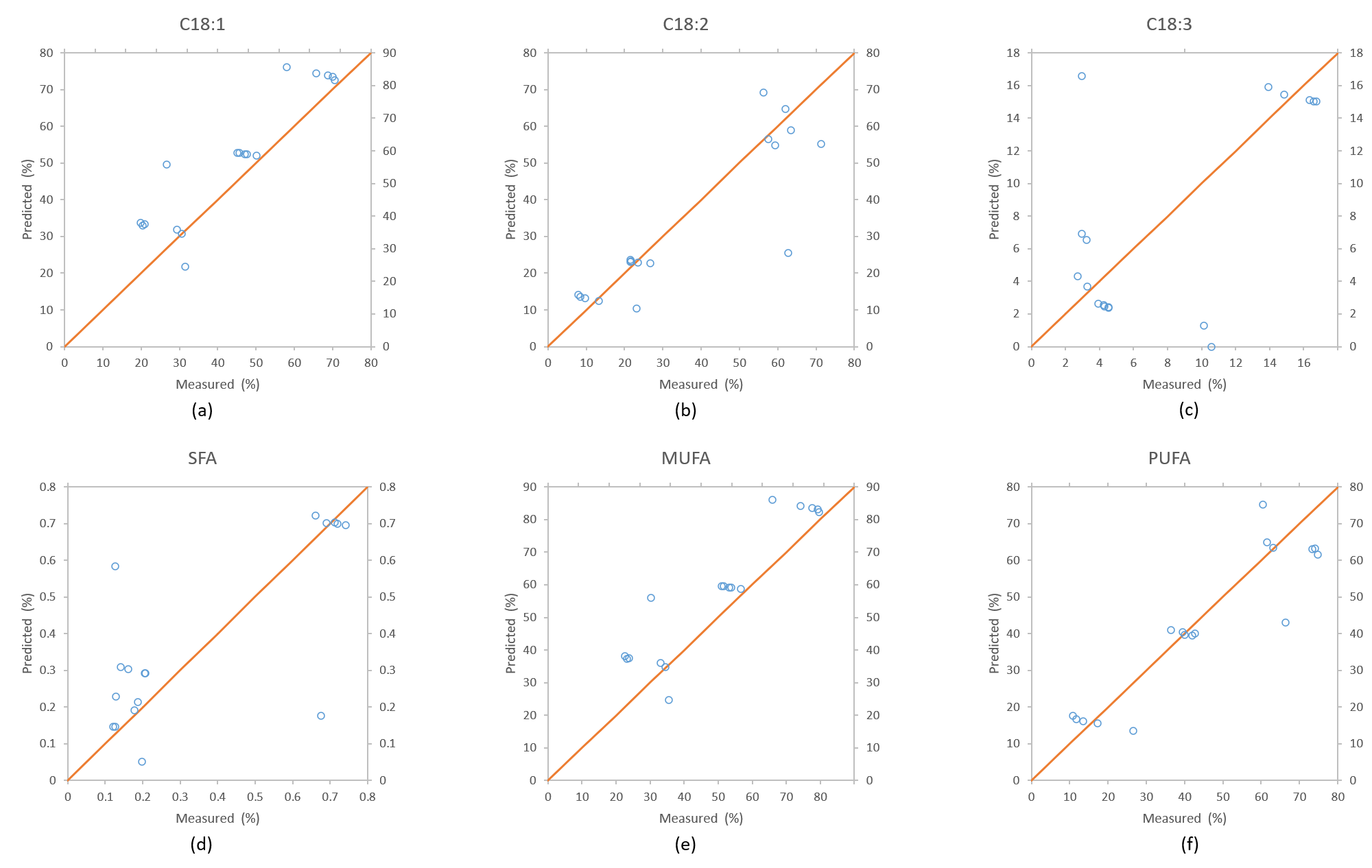


**Fig 6. Predicted versus measured for (a) C18:1, (b) C18:2, (c) C18:3, (d) SFA, (e) MUFA and (f) PUFA using PCR regression model.**


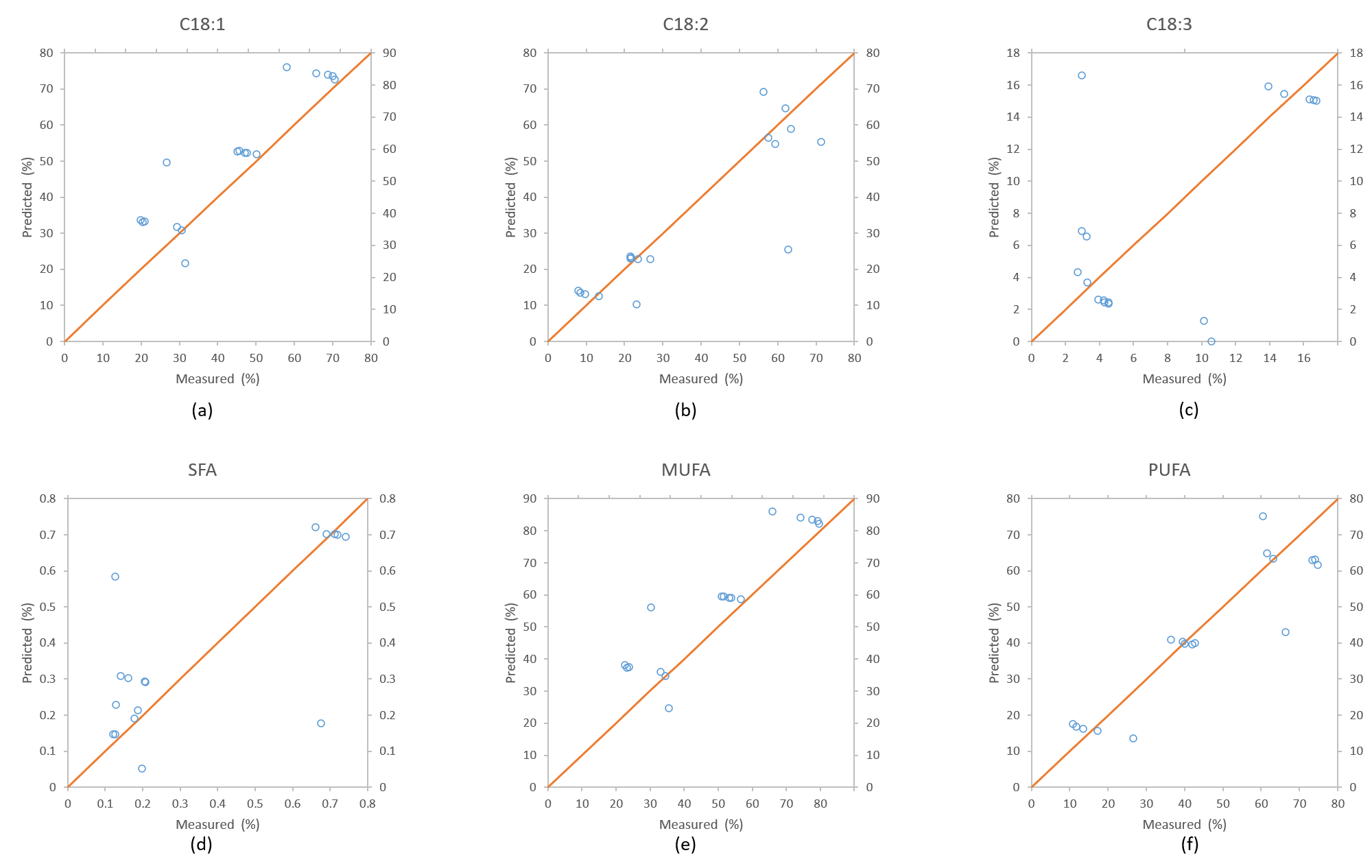


**Fig 7. Predicted versus measured for (a) C18:1, (b) C18:2, (c) C18:3, (d) SFA, (e) MUFA and (f) PUFA using PLR regression model.**
