# Supplementary material for: Analysis and prediction of the major fatty acids in vegetable oils using dielectric spectroscopy at 5–30 MHz
Source: PLoS One. 2022 May 26;17(5):e0268827. doi: 10.1371/journal.pone.0268827 (PMC9135300; doi:10.1371/journal.pone.0268827)
Supplement: Attachment — Submitted filename: PLOS Reviewer Comments_20220221_2.docx [file pone.0268827.s002.docx]

**PLOS Reviewer Comments**

| **Paper ID:** | PONE-D-21-31530 |
| --- | --- |
| **Title:** | Analysis and Prediction of the Major Fatty Acids in Vegetable Oils using Dielectric Spectroscopy at 5 – 30 MHz |

**General Comments**

| **Num** | **Comments** | **Responses** |
| --- | --- | --- |
| 1. | Please ensure that your manuscript meets PLOS ONE's style requirements, including those for file naming. The PLOS ONE style templates can be found at  <https://journals.plos.org/plosone/s/file?id=wjVg/PLOSOne_formatting_sample_main_body.pdf> and  <https://journals.plos.org/plosone/s/file?id=ba62/PLOSOne_formatting_sample_title_authors_affiliations.pdf> | The manuscript has been reconstructed as the template. |
| 2. | We understand that you purchased products from local markets for this study. In your Methods section, please provide additional regarding the source of this material. Please provide the geographic coordinates and names of the purchase locations (e.g., stores, markets), if available, as well as any further details about the purchased items (e.g., lot number, source origin, description of appearance) to ensure reproducibility of the analyses. | The geographic coordinates and names of the purchase location have been added in the document (Page 7, Line 129). We believe, the given info is enough to ensure reproducibility of the analyses. |
| 3. | Thank you for stating the following in the Acknowledgments Section of your manuscript:  “The present work was supported by the Malaysian Ministry of Higher Education under the Fundamental Research Grant Scheme [01-01-20-2247FR].”  We note that you have provided additional information within the Acknowledgements Section that is not currently declared in your Funding Statement. Please note that funding information should not appear in the Acknowledgments section or other areas of your manuscript. We will only publish funding information present in the Funding Statement section of the online submission form.  Please remove any funding-related text from the manuscript and let us know how you would like to update your Funding Statement. Currently, your Funding Statement reads as follows:  “Funding was provided by the Malaysian Ministry of Higher Education ([www.mohe.gov.my](http://www.mohe.gov.my)) through the Fundamental Research Grant Scheme under the Grant No. [01-01-20-2247FR], awarded to FZR. The funders had no role in study design, data collection and analysis, decision to publish, or preparation of the manuscript.”  Please include your amended statements within your cover letter; we will change the online submission form on your behalf. | The funding information in the Acknowledgement section was removed, and the Funding Statement section was updated. |
| 4. | In your Data Availability statement, you have not specified where the minimal data set underlying the results described in your manuscript can be found. PLOS defines a study's minimal data set as the underlying data used to reach the conclusions drawn in the manuscript and any additional data required to replicate the reported study findings in their entirety. All PLOS journals require that the minimal data set be made fully available. For more information about our data policy, please see<http://journals.plos.org/plosone/s/data-availability>.  Upon re-submitting your revised manuscript, please upload your study’s minimal underlying data set as either Supporting Information files or to a stable, public repository and include the relevant URLs, DOIs, or accession numbers within your revised cover letter. For a list of acceptable repositories, please see<http://journals.plos.org/plosone/s/data-availability#loc-recommended-repositories>. Any potentially identifying patient information must be fully anonymized.  Important: If there are ethical or legal restrictions to sharing your data publicly, please explain these restrictions in detail. Please see our guidelines for more information on what we consider unacceptable restrictions to publicly sharing data:<http://journals.plos.org/plosone/s/data-availability#loc-unacceptable-data-access-restrictions>. Note that it is not acceptable for the authors to be the sole named individuals responsible for ensuring data access.  We will update your Data Availability statement to reflect the information you provide in your cover letter. | A minimal dataset was uploaded as Supporting information |
| 5. | While revising your submission, please upload your figure files to the Preflight Analysis and Conversion Engine (PACE) digital diagnostic tool, <https://pacev2.apexcovantage.com/>. PACE helps ensure that figures meet PLOS requirements. To use PACE, you must first register as a user. Registration is free. Then, login and navigate to the UPLOAD tab, where you will find detailed instructions on how to use the tool. If you encounter any issues or have any questions when using PACE, please email PLOS at figures@plos.org. Please note that Supporting Information files do not need this step. | All figures have been uploaded and converted by PACE. |

**Reviewers’ Comments**

| **Rev.** | **Comments** | **Responses** |
| --- | --- | --- |
| 1. | Summary: The paper describes the use of dielectric spectroscopy and multivariate data analysis to determine fatty acids in vegetable oils  General comments:  The paper is in general well written. However, I have some questions regarding the experimental data and data analysis. | Thank you for the input. We hope our work can add to the existing body of scientific knowledge**.** |
|  | In the data pre-processing can the authors describe what is the mean normalization? Is it the mean center? | We have added further explanations on how mean normalization was calculated (Page 10, Line 190-193) |
|  | Figure 1- the PCA has 100% of explain variance, so why do a PCA? PCA is a data reduction method. If 100% variance is used, no data reduction occurred. | We agree with the reviewer’s comment. We have removed the explained variance from the axes of Fig. 2 (i.e., Fig. 1 in the past version) to avoid confusion (Page 13, Line 237-240, Fig.2 axes).  The PCA in Fig. 2 was used to graphically display the relation of different oil groups and the corresponding fatty acids that influence the relationship. PCA was not used as a data reduction method for the development of regression model in the later part of the paper. |
|  | My important concern is regarding the PLS analysis. First 17 samples are very few, in particularly if 5 latent variables are going to be used. Furthermore, the authors used full cross validation that is known to overfit the model, additionally, the model was not validated with external samples. This is a crucial point, therefore is my opinion that the authors need to validate the model to the paper to be published. | Prior to the administration of the study, a market survey was conducted to identify the availability of various brands of cooking oils available locally. There were 5 types/brands of olive, canola, sunflower oils, and 2 types/brands of soybean oils available in the market at the time of data collection. Other types/brands of oils were not available because these were imported products and are not commonly consumed and available, attributed to the price. We have added the explanation (Page 7, Line number 124-127). Due to limited capacity to do the experiment, we decided to proceed with one sample for each type/brand that is available locally.  In comparison to this work, *Lizhi’s et al*. (reference 26) studies the properties of different vegetable oil samples, using only 10 samples. *Dong et al*. (reference 21) predicts fatty acids composition of vegetable oil using 15 samples. |
|  | In Table 5 where are the r2 of the PLS model? Why no figure like Figure 5 was made for the PLS | The r2 of the PLS in Table 5 are very similar to the PCR model. Details of both PCR and PLS results were included in the Supporting Information.  The plot for PLS model is similar to the PCR model in Fig. 6 (i.e., Fig. 5 in the past version). Hence, we did not include it in the paper.  Details of both PCR and PLS plots were included in the Supporting Information. |
| 2. | The manuscript by Sairin et al. reports a prediction of fatty acids in vegetable oils by means of dielectric spectroscopy. The paper is well written and organized, and the significance and importance of dielectric spectroscopy study in this field is well clarified. I would like to recommend it to be published in PLOS ONE after the following points being addressed. | Thank you for the input. We hope our work can add to the existing body of scientific knowledge**.** |
|  | In the section of “dielectric spectroscopy measurement”, the authors should mention how they perform the calibration and show the calibration results by using a couple of standard liquids. | Calibration procedures detail were added, and results using distilled water, methanol and ethanol have been included (Page 9, Line 168-176) |
|  | The authors should explain why the frequency range of 5 – 30MHz is selected. Many oils could exhibit a relaxation behavior in this frequency range, if this happens, dielectric constant in this frequency range will show a decrease with increasing frequency, and the dielectric constant measured in this range cannot represent the static dielectric constant of the sample under study. | Lower frequency range (i.e. 500Hz – 1MHz) has been studied by *Lizhi’s et al. (2008).* Our work complements the current body of knowledge by covering higher frequency range (5-30MHz).  Within this selected frequency range, the results show comparable prediction performance. We have added a sentence (Page 6, Line 112-114). |
