# Supplementary material for: Analysis and prediction of the major fatty acids in vegetable oils using dielectric spectroscopy at 5–30 MHz
Source: PLoS One. 2022 May 26;17(5):e0268827. doi: 10.1371/journal.pone.0268827 (PMC9135300; doi:10.1371/journal.pone.0268827)
Supplement: Attachment — Submitted filename: PLOS Reviewer Comments_20220505.docx [file pone.0268827.s003.docx]

**PLOS Reviewer Comments**

| **Paper ID:** | PONE-D-21-31530R1 |
| --- | --- |
| **Title:** | Analysis and Prediction of the Major Fatty Acids in Vegetable Oils using Dielectric Spectroscopy at 5 – 30 MHz |

**General Comments**

| **Num** | **Comments** | **Responses** |
| --- | --- | --- |
| 1. | Please review your reference list to ensure that it is complete and correct. If you have cited papers that have been retracted, please include the rationale for doing so in the manuscript text, or remove these references and replace them with relevant current references. Any changes to the reference list should be mentioned in the rebuttal letter that accompanies your revised manuscript. If you need to cite a retracted article, indicate the article’s retracted status in the References list and also include a citation and full reference for the retraction notice. | The reference list has been reviewed and updated.  Previously reference (11) was removed [page 4 line 78], Previously reference (40) was removed and replaced with a new reference (21) [page 6 line 108] |

**Reviewers’ Comments**

| **Rev.** | **Comments** | **Responses** |
| --- | --- | --- |
| 1. | Summary: The paper describes the use of dielectric spectroscopy and multivariate data analysis to determine fatty acids in vegetable oils  General comments:  The paper was improved however there are still some minor corrections to be made. |  |
|  | Table 5 should be the one that is in supplementary material. I do not understand why the authors choose to show a “smaller” table in the paper. | Table 5 was replaced with the one in the supplementary material. Additional discussions on MAPE for PCR models were added [page 21 line 337-338, line 342] |
|  | In the conclusion section it is written that the errors are between 9.17% and 11.23%, however these errors are the RMSECV and not the MAPE. Authors should discuss the MAPE errors that are between 17.72% and 23.58%. | The errors have been changed to MAPE instead of RMSECV [page 21 line 337-338 and line 342], [page 22 line 352-353] and also in the conclusion section [page 23 line 373] |
| 2. | No response | - |
